# Structure-Based Classification and Anti-Cancer Effects of Plant Metabolites

**DOI:** 10.3390/ijms19092651

**Published:** 2018-09-06

**Authors:** Seong-Ah Shin, Sun Young Moon, Woe-Yeon Kim, Seung-Mann Paek, Hyun Ho Park, Chang Sup Lee

**Affiliations:** 1 Collage of Pharmacy and Research Institute of Pharmaceutical Sciences, Gyeongsang National University, Jinju 52828, Korea; shinsaya@gnu.ac.kr (S.-A.S.); symoon0414@gnu.ac.kr (S.Y.M.); million@gnu.ac.kr (S.-M.P.); 2 Division of Applied Life Science (BK21Plus), Plant Molecular Biology and Biotechnology Research Center (PMBBRC), Research Institute of Life Sciences (RILS), Gyeongsang National University, Jinju 52828, Korea; kim1312@gnu.ac.kr; 3 College of Pharmacy, Chung-Ang University, Seoul 06974, Korea

**Keywords:** plant, metabolite, cancer, anti-cancer drug

## Abstract

A variety of malignant cancers affect the global human population. Although a wide variety of approaches to cancer treatment have been studied and used clinically (surgery, radiotherapy, chemotherapy, and immunotherapy), the toxic side effects of cancer therapies have a negative impact on patients and impede progress in conquering cancer. Plant metabolites are emerging as new leads for anti-cancer drug development. This review summarizes these plant metabolites with regard to their structures and the types of cancer against which they show activity, organized by the organ or tissues in which each cancer forms. This information will be helpful for understanding the current state of knowledge of the anti-cancer effects of various plant metabolites against major types of cancer for the further development of novel anti-cancer drugs.

## 1. Introduction

Cancer is characterized by uncontrolled/unlimited cell growth, which can result in death [1]. Although a variety of methods to overcome and treat cancers have been researched, the number of cancer patients continues to increase each year. Furthermore, an estimated 15.5 million people in the world will become cancer patients by 2030, and 11.5 million of these cases are expected to be fatal [2]. Therefore, cancer is the leading cause of mortality and morbidity worldwide [3]. Cancers have been reported to be caused by the dysregulation of key cellular processes, such as growth signaling, anti-apoptotic signaling, immune response, gene stability, and regulation of the stromal microenvironment [1,4]. The treatment of cancer has been focused on re-regulating these cellular functions. Up to the present date, numerous clinical trials have investigated potential cures for cancer via radiation, chemotherapy, antibody treatment, and immunotherapy [5]. Radiation and chemotherapy have severe side effects due to their cytotoxicity to normal cells [3]. Antibody treatment and immunotherapy show highly specific cancer targeting ability, but have a limited target range and can be very expensive [5]. Additionally, many types of cancer tend to relapse and acquire resistance after treatment [3,5]. Currently, combination therapies involving several drugs or therapies are being used to attempt to overcome the limitations and the drawbacks of individual therapies [3,5]. Furthermore, to reduce the side effects of anti-cancer drugs and to discover more effective drugs, new approaches have been developed to identify novel molecules with anti-cancer activity from new sources [3].

Plant species have been used in medical treatment for millennia [3,4]. Additionally, plant-derived metabolites have been reported to be useful for a variety of therapeutic purposes and biotechnological applications [6]. Plant metabolites exhibit a wide range of biological functions, including anti-cancer, analgesic, anti-inflammation, and anti-microbial activities [3]. Plants have generated about 25% of clinically used drugs [7]. More than 60% of drugs with anti-cancer activity originated from plants [8]. As discussed above, the development of new molecules for cancer treatment with fewer side effects and greater efficacy is essential. Plant-derived metabolites are good sources of new anti-cancer drugs with reduced cytotoxicity and increased activity [9]. In this review, we categorize such plant metabolites according to their structure and summarize their activity according to type of cancer.

## 2. Phytochemicals as Bioactive Metabolites

Phytochemicals are constitutive metabolites that are produced by various parts of plants through their primary or secondary metabolism, and have essential functions in the plant for general growth and defense against animals, insects, microorganisms, and abiotic stress [10,11]. Primary metabolites such as carbohydrates, lipids, and proteins have a direct relationship to the growth and metabolism of the plant. Secondary metabolites, which are biosynthetically derived from primary metabolites, are not necessary for survival, but are involved in important functions in the plant, such as protection, competition, and species interactions [12,13]. These can be classified into three major groups based on their biosynthetic origins: phenolic compounds, terpenoids, and nitrogen/sulfur-containing compounds [14]. These compounds have been investigated for use in carcinomatous-related diseases, and have been reported to have diverse anti-cancer properties, such as anti-proliferation and apoptotic cell death activity. In this review, we categorize these plant metabolites according to their structure and discuss their structure and anti-cancer activity.

### 2.1. Phenolic Compounds

**Figure 1:**
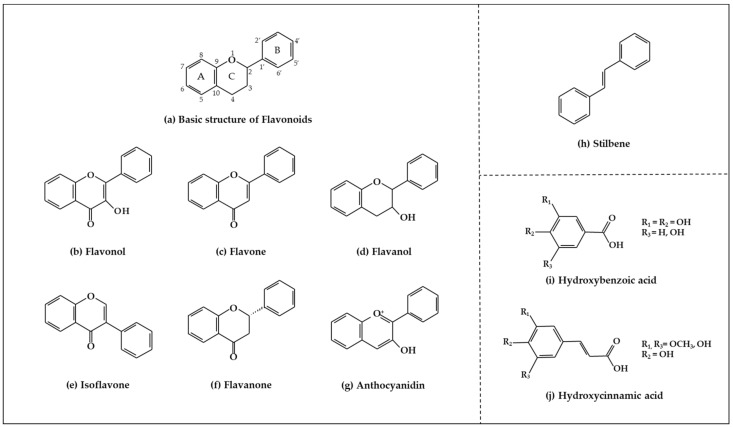
Structure of phenolic compounds.

Phenolic compounds (Figure 1), a type of plant secondary metabolites, are polyhydroxylated phytochemicals found in plant, fruits, vegetables, spices, nuts, and grains [15]. They are one of the most abundant and widely distributed groups of natural compounds available to human beings [16]. Secondary metabolites with phenolic structures play key roles in various ecological relationships between plants and other living things and their physical environment [15,16]. The structures of polyphenol compounds are characterized by at least one aromatic ring with one or more hydroxyl groups [17]. They are categorized by the structural components binding these rings to one another, and by the number of phenol rings that they contain. Polyphenolic compounds are believed to have anti-cancer activity, and include flavonoids, stilbenes, and phenolic acids [18].

#### 2.1.1. Flavonoids

Flavonoids (Figure 1a) are the largest and most diverse sub-group of polyphenolic compounds that are produced as plant secondary metabolites [19]. These compounds are found in various fruits and vegetables, including several medicinal plants, and they also have critical roles in the growth, development, and defense of plants [19]. The basic structure of flavonoids consists of two benzene rings (A and B) linked by a heterocyclic ring (C) with a carbon bridge [20]. Most of the more than 6,000 flavonoids that have been identified from a variety of plants can be categorized into the flavonol, flavone, flavanol, isoflavone, flavanone, or anthocyanidin subclasses according to their structure [21].

Flavonols (Figure 1b) are the most ubiquitous subclass of flavonoids, and are found in plants and fruits such as olives, onions, kale, apples, beans, and green leaves [22]. The main representatives of this subclass are quercetin, kaempferol, myricetin, isorhamnetin, and rutin. Flavonols have a hydroxyl group (-OH) on the 3-position of the C-ring. These hydroxyl groups are present in a glycosylated form in plants in combination with a sugar (commonly glucose or rhamnose) [23,24]. The biological activities of flavonols have been reported to play an important role in preventing carcinogenesis through anti-proliferation, anti-oxidation, and apoptosis activity in various cancer cell lines [25]. 

Flavones are mainly found in fruits, spices, and vegetables such as celery, olives, onion, garlic, citrus fruits, pepper, and parsley [22,23]. Although the flavone 2-phenyl-4*H*-1-benzopyran-4-one is the core structure of flavonoids, flavones are much less common than flavonols among plant metabolites [26]. Flavones (Figure 1c) are present chiefly as 7-*O*-glycosides. They are mainly present in forms such as luteolin and apigenin, while less abundant flavones include tangeretin, nobiletin, baicalein, wogonin, and chrysin [23]. The chemical structure of these flavones consists of a 3-hydroxyflavone backbone, which is the simplest flavone structure, and may contain a broad range of functional groups, including hydroxyl groups, carbonyl groups, and conjugated double bonds [18]. Flavones have been reported to have a variety of biological activities, including antioxidant, anti-proliferative, anti-tumor, anti-microbial, estrogenic, acetyl cholinesterase, and anti-inflammatory activities, and are used for controlling various types of disease, such as cancer, cardiovascular disease, and neurodegenerative disorders [26].

Flavanols, which are sometimes referred to as flavan-3-ols, are derivatives of flavans (Figure 1d). Flavanols have a hydroxyl group at the C3 position [27]. They are the most varied and complex subgroup of flavonoids, and exist in states ranging from single molecules to oligomers, polymers, and other derivatives [28]. Flavanol compounds include catechin, epicatechin, epicatechin-3-*O*-gallate, theaflavins, epigallocatechin-3-*O*-gallate, proanthocyanidins, and thearubigins [27,29]. Moreover, they are present in fruits and vegetables such as pears, green leaves, berries, cherries, red grapes, currants, and apples [30]. The flavanols have been reported to exhibit several biological activities such as anti-oxidation, anti-carcinogenesis, cardioprotective, and anti-viral effects [31]. However, most flavanol-related data has been derived from medium/small-scale and short-term (from weeks to several months) dietary intervention studies [32].

Isoflavones (Figure 1e) are secondary metabolites of flavonoids that occur naturally in members of the *Leguminosae/Fabaceae* family, such as kudzu, lupine, soybeans, red clover, peanuts, chickpeas, broccoli, cauliflower, barley, fava beans, and alfalfa [33,34]. The benzene ring (B) of isoflavones is linked to C3 of the heterocyclic ring by a carbon bridge. The isoflavone compounds include genistein, daidzein, biochanin A, glycitein, and formononetin [34]. Isoflavones are also classified as phytoestrogens because of their structural similarities with estrogen, particularly 17-β-estradiol (a human female hormone), and can bind to both alpha and beta estrogen receptors [24,33,35]. Therefore, they can exert various bioactivities in some hormone-dependent diseases by modulating the expression of genes that control cell survival [35,36].

Flavanones (Figure 1f) are non-planar flavonoids that are derived chiefly in mono- and di-glycoside forms, but are less frequently present in aglycone form [23]. Although flavanones are found in tomatoes and selected aromatic plants such as mints, they are almost exclusively present in high concentrations in citrus fruit [24]. The most common flavanone glycosides, which are generally glycosylated by a disaccharide, are neohesperidin, naringenin, and hesperetin [18]. These glycosides are abundant in the fruit of oranges, grapefruit, and tomatoes, and also found in the peels of citrus, bitter oranges, and grapefruit [37,38]. 

Anthocyanins (glycosylated forms of anthocyanidin (Figure 1g)) are polyphenolic pigments that belong to the water-soluble flavonoid group, and impart red, blue, and purple colors to plants in a pH-dependent manner [39,40]. They are found in plant organs such as fruits, flowers, and leaves, including those of grapes, berries, pomegranate, red cabbage, purple corn, apples, radishes, tulips, roses, and orchids [39]. More than 700 anthocyanin derivatives have been verified in nature [41]. Anthocyanins vary in their number of hydroxyl groups and the degree of methylation of the aglycone molecule. Additionally, the number and the location of sugars connected to the aglycone molecule, and the number and the character of aliphatic or aromatic acids connected to these sugars, can also vary [23,42]. The most abundant anthocyanins are cyanidin, peonidin, pelargonidin, delphinidin, petunidin, and malvidin [43]. Although anthocyanins are non-essential nutrients, they may promote the maintenance of health and can confer protection against chronic diseases [41]. Recently, research into anthocyanins has been highlighted due to their potential preventative and/or therapeutic effects for a variety of diseases [40].

#### 2.1.2. Stilbenes

Stilbenes (Figure 1h) are a class of nonflavonoid polyphenol phytochemicals [18]. Their molecular backbone consists of 1,2-diphenylethylene units. Stilbenes can be categorized as monomeric and oligomeric stilbenes [44]. These compounds are somewhat limited in plants, since the core enzyme in stilbene biosynthesis, stilbene synthase, is not universally expressed [45]. However, due to their bioactive properties and low toxicity, stilbenes have a remarkable potential for the prevention and treatment of a variety of diseases, including cancer [46,47]. The most representative stilbene derivatives are the stilbenoids, which are hydroxylated derivatives of stilbene that can act as phytoalexins. Such compounds include resveratrol, pterostilbene, gnetol, and piceatannol, and are derived from grapes, berries, peanuts, and other plant sources [45,46]. Among these, resveratrol is the most widely studied stilbenoid. Resveratrol is found as cis- and trans-isomers, as well as conjugated derivatives (*trans*-resveratrol-3-*O*-glucoside) [18]. In addition, resveratrols have been reported to show cancer chemopreventive properties by blocking carcinogenesis [48,49,50].

#### 2.1.3. Phenolic Acids

Phenolic acids are secondary metabolites that are present in almost all plant-derived foods including mushrooms, berries, black currants, kiwis, plums, apples, pears, chicory, and potatoes [30,51]. These compounds can be classified into two major groups, hydroxybenzoic and hydroxycinnamic acids, which are derived from the non-phenolic benzoic and cinnamic acids [51]. The most common hydroxybenzoic acids (Figure 1i) are gallic, p-hydroxybenzoic, syringic, vanillic, and protocatechuic acid, while the corresponding hydroxycinnamic acids (Figure 1j) are caffeic, chlorogenic, coumaric, ferulic, and sinapic acid [24]. These compounds are present in both free and bound forms in all plant-derived foods. The bound forms are most frequently esters, glycosides, and bound complexes [52]. Phenolic acids have been reported to have powerful antioxidant properties and biological activities including cardioprotective, anti-carcinogenic, antimicrobial, and hepatoprotective properties [53].

### 2.2. Terpenoids

**Figure 2:**
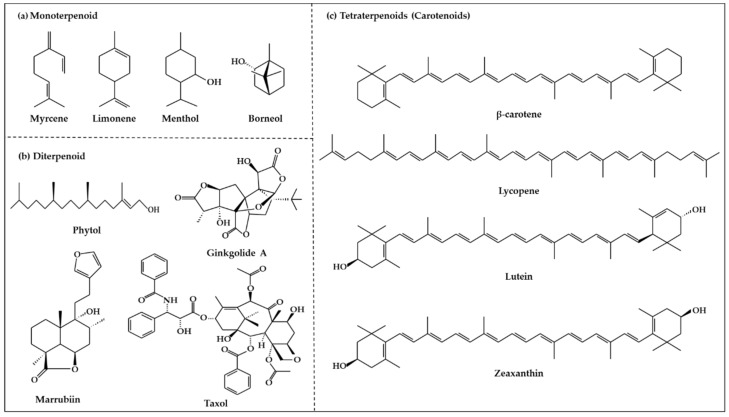
Structure of terpenoids.

Terpenoids (Figure 2), which are also known as isoprenoids, are one of the most numerous and structurally diverse classes of metabolites [54]. They are flammable non-saturated hydrocarbons that exist in the liquid state, and are typically found in essential oils, resins, or oleoresins [55]. Terpenoids are based on linear arrangements of isoprene, and their carbon skeletons consist of two or more carbon units [56,57]. In particular, terpenoids can be classified as mono-, di-, or tetraterpenoids based on isoprenoid biosynthesis in the plastid [18].

#### 2.2.1. Monoterpenoids

Monoterpenoid structures comprise two isoprene units (C10) and can be divided into three sub-groups: acyclic, monocycles, and bicycles (Figure 2a) [56]. The monoterpenoids within each group are simple unsaturated hydrocarbons and can have functional groups such as alcohols, aldehydes, and ketones [56]. The most important representatives are myrcene, citral, linalool, α-terpineol, limonene, thymol, menthol, carvone, eucalyptol, α/β-pinene, borneol, and camphor [58]. Monoterpenoids can be isolated from the fragrant oils of many plants, and are also found in many marine organisms, where they are generally halogenated. In addition, they are well known as components of the essential oils of flowers and herbs, pollinator attractants, and defense compounds [18]. Moreover, monoterpenoids have been reported to potentially act as antioxidants and are widely used as medicines with antimicrobial, antiseptic, disinfectant, and wound-healing properties [59].

#### 2.2.2. Diterpenoids

Diterpenoids constitute a large group of compounds derived from geranylgeranyl pyrophosphate (Figure 2b) [18]. Their structure comprises a C20 carbon skeleton based on four isoprene units [56], and they can be classified into linear, bicyclic, tricyclic, tetracyclic, pentacyclic, or macrocyclic subgroups based on their skeletal core [58]. Diterpenoids are present in higher plants, fungi, insects, and marine organisms [57]. They are typically found in polyoxygenated form with ketone and hydroxyl groups [56]. Typical compounds of this group include phytol, sclareol, marrubiin, salvinorin A, abietic acid, 9-geranyl-α-terpineol, gibberellin A1, ginkgolide A, and taxol [18,60]. Diterpenoids have been reported to have cytotoxic and anti-proliferative properties [61].

#### 2.2.3. Tetraterpenoids (Carotenoids) 

Tetraterpenoids consist of eight isoprene units and have a 40-carbon backbone [56]. Carotenoids (Figure 2c)*,* the most common class of tetraterpenoids, are a group of natural pigments produced in plants, algae, bacteria, and fungi [62]. They are the key source of the yellow, orange, and red colors in many plants, including the orange-red colors of oranges, tomatoes, and carrots and the yellow colors of many flowers [62,63]. Carotenoids are essential both in plants and animals. However, they cannot be synthesized in animals, and therefore must be obtained from dietary sources. In addition, carotenoids are known to have protective activity against some forms of cancer, particularly lung cancer [64]. Their beneficial effects are thought to be due to their role as antioxidants [65]. Based on their chemical structure, carotenoids can be generally classified into two classes, carotenes and xanthophylls [58]. Carotenes are non-oxygenated carotenoids that may be linear or possess cyclic hydrocarbons, and include β-carotene, α-carotene, and lycopene [56]. Xanthophylls are the oxygenated derivatives of carotenes, and include β-cryptoxanthin, lutein, zeaxanthin, meso-zeaxanthin, astaxanthin, and canthaxanthin [64]. Carotenoids play a critical role in various biological processes such as the immune response, prevention of cell propagation, induction of apoptosis, and suppression of several cancers [66,67]. Therefore, carotenoid deficiency can cause health problems in human beings.

### 2.3. Nitrogen-Containing Alkaloids and Sulfur-Containing Compounds

#### 2.3.1. Alkaloids

**Figure 3:**
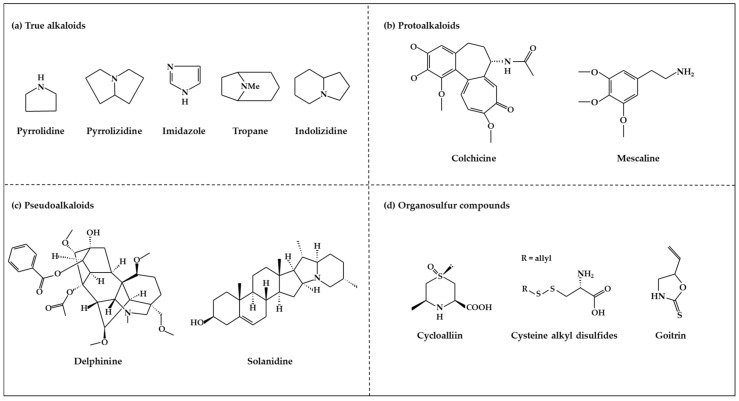
Structure of nitrogen-containing alkaloids and sulfur-containing compounds.

Alkaloids are secondary metabolites containing a basic nitrogen, and are found primarily in plants [68]. The most common forms are derived from amino acids, whereas others originate from the modification of various classes of molecules such as polyphenols, terpenes, or steroids [14]. Alkaloids are produced by a large variety of organisms including bacteria, fungi, and animals [69]. Alkaloids have diverse biological functions, including anti-cancer, anti-microbial, anti-inflammatory, and antinociceptive properties [70]. Therefore, they play roles as protective agents against various diseases [70,71]. Individual plant species produce only a few kinds of alkaloids [68]. Certain plant species, such as *Papaveraceae*, *Ranunculaceae*, *Solanaceae*, and *Amaryllidaceae,* are particularly rich in alkaloids [68,72]. Although there is no uniform classification scheme for alkaloids, they can be generally divided into the following major groups: true alkaloids, protoalkaloids, and pseudoalkaloids [69,73]. True alkaloids (Figure 3a) are derived from amino acids, and have a nitrogen-atom-containing heterocyclic ring [74]. This group is further divided into 14 sub-groups according to the ring structure: pyrrolidine, pyrrolizidine, piperidine, tropone, quinoline, isoquinoline, acridine, quinolizidine, benzopyrrole, indolizidine, imidazole, purine, quinolizidine, and oxazole. The second group, protoalkaloids (Figure 3b), are derived from amino acids but do not contain a nitrogen-atom-bearing heterocyclic ring. These are less commonly found in nature in comparison with true alkaloids. The protoalkaloids include hordenine, mescaline, ephedrine, colchicine, erythromycin, jurubin, pachysandrine A, and taxol. Finally, although pseudoalkaloids (Figure 3c) are not derived from amino acids, they contain a nitrogen atom in a heterocyclic ring, and include subclasses such as terpene- and steroid-like alkaloids: delphinine, aconitine and solanidine [69,73].

#### 2.3.2. Organosulfur Compounds

Organosulfur compounds (OSC) are sulfur-containing organic compounds (Figure 3d) [75]. Some essential amino acids and enzymes, sulfides, disulfides, and other OSCs are generated in the bodies of all living creatures and the natural environment [75,76]. OSCs can both maintain normal health in the human body and contribute to the development of disease by determining the thiol/disulfide redox states in body [75,77]. There are two major groups of vegetables that contain OSCs with special properties [75,76]. One is the *Allium* genus (family *Amaryllidaceae*), which produces *S*-alk(en)yl-l-cysteine sulfoxides, and includes plants such as garlic, onions, shallots, leeks, and chives. The second group includes members of the *Brassica* genus, including cabbage, cauliflower, Brussels sprouts, and kale and the members of the *Eruca* genus of the mustard or cruciferous family, which includes plants such as rucola; this group contains S-methyl cysteine-l-sulfoxide. The OSCs of vegetables from the *Allium*, *Eruca*, and *Brassica* genera include cycloalliin, thiosulfonates, cysteine alkyl disulfides, glucosinolates, goitrin, and epithionitrile [75,76]. There is an abundance of epidemiological and experimental evidence that indicates that OSCs have protective effects against several cancers, including breast cancer [75,76].

## 3. Anti-Tumor Activity of Plant Metabolites in Various Malignant Cancers

### 3.1. Colorectal Cancer

Colorectal cancer is the major cause of cancer-mediated death worldwide. Nutrients and food play an important role in the development of colorectal cancer, and eating mostly food of plant origin rather than red and processed meat is recommended for cancer prevention [6]. Secondary metabolites from potatoes have been found to inhibit the growth of colon cancer cells [78]. The maximum cancer cell growth inhibition was achieved when HT-29 colon cancer cells were exposed to extracts of potatoes with red-and purple-fleshed tubers. This indicates that some metabolites of potatoes with red and purple tubers could be valuable as a dietary intervention against developing the colon cancer [79]. Dichamanetin, a secondary metabolite from *Piper sarmentosum*, which is an edible herb used as a spice in Southeast Asia, was reported to reduce cell viability in HT-29 colon cancer cells [80]. This metabolite showed dose-dependent cytotoxic effects on this cancer cell type via the induction of ROS, and also arrested their cell cycle, suggesting that it could be used to block cancer cell proliferation [80]. Active oxyprenylated natural products from citrus fruits belonging to the *Rutaceae* family have been considered as interesting phytochemicals for several decades [81]. For example, 4′-geranyloxyferulic acid (GOFA) has been reported to have chemopreventive activity against cancer since it was first extracted in 1966 from *Acronychia baueri* Schott (Fam. *Rutaceae*) [82]. 3-(4′-Geranyloxy-3′;-methoxyphenyl)-l-alanyl-l-proline (GAP), a peptide prodrug of GOFA, was discovered to suppress colitis-related carcinogenesis in the colon in the azoxymethane (AOM)/dextran sodium sulfate (DSS)-induced cancer model in animals [83]. Similarly, GOFA/β-CD (the β-cyclodextrin inclusion compound of GOFA) inhibited the development of colonic carcinoma in the AOM/DSS model [84]. Auraptene (7-geranyloxycoumarin, AUR), one of the lead anti-cancer compounds from the *Rutaceae* family, was also found to inhibit the growth of both wild-type and chemo-resistant colon cancer cells and suppress the formation of colonospheres, suggesting that it could prevent the recurrence of cancer stem cells [85]. Curcumin is the main secondary metabolite derived from *Curcuma longa* and other *Curcuma spp*, and has been widely studied as a therapeutic agent having antiangiogenic, anti-inflammatory, and antioxidant activity [86]. Recently, curcumin was found to suppress the oncogenicity of human colon cancer cells by reducing the stability of SIRT1 (a NAD^+^ dependent histone/protein deacetylase) and to suppress the growth of HCT-116 tumor xenografts [87]. Genistein, a phenolic compound found in soybeans, is known to act as a chemopreventive agent against various tumors [88]. 

This had inhibitory effects on colorectal cancer cells HCT 116 and LoVo; it inhibited cell proliferation and induced apoptosis [89,90]. It also inhibited the invasion and migration of colorectal cancer cells and inhibited the metastasis of human colorectal cancer cells implanted in nude mice [91]. Combinatorial treatment of genistein and indole-3-carbinol synergistically induced apoptosis of HCT 116 cells [92]. Benzyl isothiocyanate (BITC), an organosulfur compound, suppressed the viability of HCT 116 cells and activated the PI3K/Akt/forkhead box O pathway, which influences drug resistance in various human cancer cells [93]. A combination treatment with an inhibitor of the PI3K/Akt/forkhead box O pathway potentiated cell death of colorectal cancer cells induced by BITC [93]. Sulforaphane, another isothiocyanate, has an anticancer effect on the human colon cancer cell line HT-29. It downregulates the expression of microsomal prostaglandin E synthase-1, which is involved in the synthesis of prostaglandin E2 known to be highly expressed in colorectal cancer [94]. Dietary phenethyl isothiocyanate (PEITC) improved adenocarcinoma in azoxymethane (AOM) and dextran induced colitis associated cancer mouse models [95]. Recently, 6-(methylsulfinyl)hexyl isothiocyanate (6-MSITC), obtained from *Wasabia japonica*, was found to induce apoptosis in human colorectal cancer cells ( HCT 116 *p53*^+/+^ and HCT 116 *p53^−^*^/*−*^ ) via *p53*-independent mitochondrial dysfunction [96].

### 3.2. Gastric Cancer

Gastric cancer, also known as stomach cancer, is one of the most common cancers, and has a poor prognosis [97]. Although many other factors contribute to gastric tumorigenesis, there is strong evidence that *H. pylori* infection is the predominant etiological factor in the induction of gastric cancer [98]. Many plant phytochemicals used as anti-gastric-cancer agents have been found to not only affect cancer cells directly but also to inhibit *H. pylori*. Resveratrol (3,4,5′-hydroxystilbene), a polyphenol flavonoid, is known to be produced by a limited number of plants (about 31 genera), and has the ability to inhibit *H. pylori* growth and the proliferation of gastric cancer cells [99]. Isothiocyanates (ITCs) are phytochemicals derived from cruciferous plants, including allyl isothiocyanate, sulforaphane (SFN), benzyl isothiocyanate (BITC), and phenethyl isothiocyanate (PEITC). ITCs have been reported not only to have bactericidal activity toward *H. pylori* and to reduce the colonization of *H. pylori* in the stomach, but also to have chemopreventive effects on gastric cancer in vitro and in vivo [100]. SFN was found to eradicate extracellular and intracellular *H. pylori* and block benzo[a]pyrene-induced stomach tumors in mice [101]. PEITC induced cell cycle arrest and apoptosis by disrupting microtubule filaments in MKN74 and Kato-III human gastric cancer cells [102]. Another group demonstrated that PEITC reduced the invasion and the migration of AGS human gastric cancer cells through blocking the mitogen-activated protein kinase (MAPK) signaling pathways that regulate the expression of matrix metalloproteinases (MMPs)-2 and -9 [103]. BITC was also found to inhibit the migration and invasion of AGS human gastric cancer cells in a dose-dependent manner [104]. In addition to colorectal cancer, curcumin has also been extensively investigated for its chemopreventive effects on gastric cancer. In an in vitro study, curcumin was shown to inhibit the proliferation of SGC-7901 human gastric cancer cells by facilitating the collapse of the mitochondrial membrane potential, and in an in vivo study, the growth of xenograft tumors was reduced by curcumin [105]. In addition, another in vivo study showed that curcumin reduced lymphatic vessel density (LVD) in gastric-tumor bearing nude mice [106]. Quercetin, a natural flavonoid present in various fruits, was reported to induce apoptosis in BGC-823 human gastric cancer cells [107]. Recently, a combined treatment with curcumin and quercetin was found to significantly inhibit proliferation and induce apoptosis in BGC-823 cells [108]. Allicin, an active compound derived from garlic, was found to have chemopreventive effects on gastric cancer by inhibiting cell growth, arresting the cell cycle, and inducing apoptosis [109]. 

### 3.3. Lung Cancer

Lung cancer is the most common cancer, and has the highest cancer-related mortality worldwide [110]. Several secondary metabolites have been discovered to have inhibitory activity against lung cancer. Epigallocatechin gallate (EGCG), a major component of green tea from *Camellia sinensis*, has been reported to have preventive effects on carcinogenesis [111]. There are several reports that EGCG can inhibit lung cancer in vitro. Recently, EGCG was shown to inhibit the growth of several types of human lung cancer cells via upregulating p53 expression, increasing p53 stability, and inhibiting p53 ubiquitination [112]. Another study indicated that EGCG was involved in increasing miR210, a major miRNA (micro RNA) regulated by HIF-1α, resulting in a significant reduction of the proliferation and growth of mouse and human lung cancer cells [113]. Liu et al. reported that EGCG inhibited not only TGF-β-induced cell migration and invasion but also TGF-β-induced epithelial-to-mesenchymal transition (EMT) via inhibition of the Smad2 and ERK1/2 signaling pathways in nonsmall cell lung cancer (NSCLC) cells [113]. EGCG has also been found to inhibit telomerase and induce apoptosis in both drug-sensitive and drug-resistant small cell lung cancer (SCLC) cells [114]. 

In addition to their activity against gastric cancer discussed above, ITCs have also been reported to have anti-lung cancer activity via various molecular mechanisms [111]. There are three different types of ITCs [115]: BITC, PEITC, and SFN. All three ITCs arrested the growth of human lung cancer A549 cells by binding to tubulin, with their relative activities following the order BITC > PEITC > SFN [115]. BITC inhibited the growth of NSCLC cells that are resistant to gefitinib, which is widely used in treatment of NSCLC, via cell cycle arrest and reactive oxygen species generation [116]. BITC was also reported to inhibit tumorigenesis of A/J mice induced by the polycyclic aromatic hydrocarbons (PAHs) found in cigarette smoke [117]. In addition, PEITC induced the apoptosis of NSCLC cells by inducing the disassembly of actin stress fibers and degradation of tubulin, resulting in the inhibition of NSCLC cell growth [118]. In another study, both BITC and PEITC were shown to induce the apoptosis of highly metastatic lung cancer L9981 cells by activating three mitogen-activated protein kinases (MAPKs): JNK, ERK1/2, and p38 [113]. Oral SFN treatment of mice with lung cancer induced by benzo(a)pyrene (B(a)P) was proved to rehabilitate carcinogenic lungs via decreasing H_2_O_2_ production and inducing apoptosis [119]. Combination treatment with SFN and tumor necrosis factor-related apoptosis-inducing ligand (TRAIL) induced apoptosis in A549 lung adenocarcinoma cells, which are resistant to the apoptotic effect of TRAIL, through downregulation of ERK and Akt [120].

Indole-3-carbinol (I3C) is a hydrolysis product of glucosinolate, which is a natural component in members of the *Brassica* family including broccoli, cabbage, cauliflower, and Brussels sprouts, and is known to have various anti-tumor activities [111]. I3C has lung cancer-preventive activity during the progression of tobacco carcinogen induced lung adenocarcinoma in mice and is involved in the modulation of apoptosis-related proteins in lung cancer A549 cells [121]. Choi et al. showed that I3C induced cell cycle arrest at the G0/G1 phase through increasing the expression of phosphorylated p53 and cyclin D1 and activated caspase-8 mediated apoptosis via increasing Fas mRNA in lung cancer A549 cells [122]. The anti-lung cancer activity of I3C in combination with silibinin, the major active constituent of *Silybum marianum*, is stronger than that of single treatment and avoids undesirable side effects in A549 and H460 lung cancer cells and in vivo 4-(methylnitrosamino)-1-(3-pyridyl)-1-butanone (NNK)-induced lung tumors [123]. The overexpression or underexpression of microRNAs (miRNAs), which function as tumor suppressors, during tumorigenesis has been studied. It has been reported that I3C can inhibit carcinogenesis by modulating the expression of several miRNAs in the vinyl carbamate (VC)-induced lung cancer model [124].

Genistein inhibits SCLC cell proliferation and migration and induces apoptosis in the SCLC cells H446 through downregulation of FoxM1, whose target genes regulate the cell cycle and apoptosis [125]. Several reports have also indicated that genistein has synergistic effects with other well-known anti-cancer drugs. The combination of genistein with gefitinib, a drug widely used in the treatment of various cancers, can inhibit cell proliferation and induce apoptosis in drug resistant H1975 NSCLC cells, which harbor an epidermal growth factor receptor (EGFR) mutation [126]. Another report showed that treating H460 lung cancer cells with a combination of genistein and the chemotherapeutic agents cisplatin, docetaxel, or doxorubicin inhibited cell growth and induced apoptosis with greater anti-cancer activity than single treatment alone. Furthermore, genistein can inhibit the induction of nuclear factor kappaB (NF-κB) activity by chemotherapeutic agents, which enables cancer cells to become drug resistant [127].

Fisetin (3,3′,4′,7-tetrahydroxyflavone) is a polyphenolic flavonoid found in many fruits and vegetables, and has been reported to possess anti-inflammatory, antiangiogenic, and anti-tumor activities [128]. It has dual inhibitory effects on phosphatidylinositol-3 kinase (PI3K)/Akt and the mammalian target rapamycin (mTOR) signaling in A549 human NSCLC cells and inhibits the cell viability and colony-forming ability of A549 cells [129]. Fisetin is also involved in inhibiting the invasion and migration of A549 NSCLC cells through the inactivation of the extracellular signal-regulated kinase (ERK) signaling pathway and reducing the expression of MMP-2 and urokinase-type plasminogen activator (u-PA) [130]. Orally administered fisetin inhibits lung carcinogenesis by alleviating mitochondrial dysfunction and inducing apoptosis in the B(a)P-induced lung cancer mouse model [131]. In another in vivo study, fisetin inhibited angiogenesis and tumor growth in Lewis lung carcinoma bearing mice, and the combination of fisetin with cyclophosphamide (CPA), a medication used as chemotherapy, showed markedly improved anti-tumor activity over fisetin or CPA alone without toxic side effects [132].

Punicalagin (PC) is an ellagitannin, a type of phenolic compound found in *Punica granatum* (pomegranate), which has been shown to exert antioxidant, anti-mutagenic, and anti-cancer activity [133]. PC has anti-mutagenic potential and shows dose-dependent anti-proliferative effects in A549 and H1299 human lung cancer cells [134]. Pomegranate fruit extracts (PFE) inhibit not only the growth and viability of A549 lung cancer cells in vitro but also the growth of A549 lung cancer cells in nude mice in vivo [135]. Additionally, PFE has been reported to inhibit tumorigenesis in the B(*a*)P-induced lung cancer mice model [136].

Curcumin has also been reported to have anti-cancer activity in both NSCLC and SCLC cell lines [111]. In NSCLC cells, curcumin inhibits cell growth and invasion by suppressing the expression of Metastasis-associated protein 1 (MTA1) and subsequently inactivating the Wnt/β-catenin pathway, which has a cooperative role in promoting lung tumorigenesis [137]. Curcumin downregulates the expression of Cdc42, which is known to be involved in the proliferation, metastasis, and invasion of cancer cells, resulting in inhibition of the invasion of lung cancer cells [126]. One of the underlying mechanisms for the inhibition of lung cancer cell growth by curcumin was the induction of autophagy via activating the AMP-activated protein kinase (AMPK) signaling pathway [138]. In addition, curcumin is involved in lowering the resistance of NSCLC cells against erlotinib, a drug used for NSCLC [139]. In SCLC cells, curcumin suppressed cell proliferation, migration, invasion, and angiogenesis through inhibiting the signal transducer and activator of transcription 3 (STAT3) and downregulating the expression of STAT3-regulated gene products (Cyclin B1, Bcl-X_L_, survivin, vascular endothelial growth factor, MMP-2, -7, and intercellular adhesion molecule-1) [140]. Curcumin-induced apoptosis was accompanied by mechanisms that increased the intracellular reactive oxygen species (ROS) level [141].

### 3.4. Breast Cancer

Breast cancer represents the most common and highest-mortality malignancy in females around the world [142]. Naturally occurring compounds have been studied for their chemopreventive effects on breast cancer. Tomatine is a glycoalkaloid secondary plant metabolite occurring in the *Solanaceae* family of plants that is known to have defensive activities against phytopathogens [143]. It can also induce cell cytotoxicity and apoptosis and decrease metastasis-related MMP-2, -9 activity in MCF-7 human breast cancer cells [144].

I3C shows effective anti-tumor properties in estrogen receptor α (ERα)-positive breast cancer cells through the ligand-activated aryl hydrocarbon receptor (AhR), which amplifies ERα signaling via ROS induction by the upregulation of cyclic-AMP-dependent transcription factor (ATF)-3 and downstream pro-apoptotic BH3-only proteins [145]. Also, I3C inhibits tumor sphere formation in breast cancer cells with stem/progenitor cell-like character by selectively stimulating the interaction of nucleostemin (a cancer stem/progenitor cell marker highly expressed in breast cancer stem cells) with MDM2 (an inhibitor of p53 tumor suppressor) [146].

Triterpenoids are secondary metabolites found in various plants, and are known to have antioxidant, anti-microbial, anti-allergic, and anti-angiogenic activity. Dozens of triterpenoids have been reported to have chemopreventive potential against breast cancer [147]. Curcubitane-type triterpenoids isolated from *Cucurbitaceae* family inhibit the growth of several types of human breast cancer cells [148,149,150], exhibit cytotoxicity against these cells [151,152], and induce apoptosis [153,154,155]. Dammarane triterpenoids isolated from the tropical plant *Chisocheton penduliflorus* exhibit weak cytotoxicity in breast cancer cells [156]. Two major friedelane triterpenoids, pristimerin and celastrol, have been found to be active against breast cancer cells. Pristimerin acts as a mitochondrial-targeting compound and induces caspase-mediated apoptosis and cytochrome *c* release in MDA-MB-231 breast cancer cells [157]. Celastrol has been shown to not only inhibit the growth and induce apoptosis of W256 rat breast cancer cells, but also suppress their migration by acting as an inhibitor of IκB kinase (IKK) [158]. Meliavolkenin, a limonoid triterpene isolated from *Melia volkensii* (Meliaceae), has cytotoxic effects on MCF-7 breast cancer cells [159]. Betulinic acid (BA), a pentacyclic triterpenoid, has anti-proliferative activity in MCF-7 and T47D breast cancer cells [160], in which a decrease in bcl2 and cyclin D1 gene expression and an increase in the bax gene were also observed [161]. In another study, most breast cancer cell lines (SKBR3, MDA231, MDL13E, BT483, BT474, T47D, and BT 549) except for MCF7 and ZR-75-1 cells were sensitive to BA treatment [162]. Lupeol, another natural pentacyclic triterpenoid, inhibits proliferation in estrogen receptor alpha (ERα)-negative MDA-MB-231 cells [163]. Ursolic acid, a pentacyclic triterpenoid widely found in the peels of fruits, has been studied as a potential inhibitor of breast tumors. Ursolic acid inhibits MCF-7 cell proliferation through arresting the cell cycle at G1 [164] and possesses cytotoxic activity against MCF-7 and MDA-MB-231 cells [165,166,167]. Additionally, ursolic acid is involved in inducing apoptosis through modulation of the glucocorticoid receptor (GR) and Activator protein 1 (AP1) in MCF-7 cells [168]. Yeh et al. observed that it has suppressing effects on migration and invasion through inactivation of c-Jun N-terminal kinase (JNK), Akt, and mTOR signaling in highly metastatic MDA-MB-231 breast cancer cells [169]. Another pentacyclic triterpenoid, asiatic acid, which is extracted from the tropical medicinal plant *Centella asiatica*, was found to inhibit cell growth by inducing S-G2/M phase cell cycle arrest and executing apoptosis through the activation of mitochondrial pathways in MCF-7 and MDA-MB-231 cells [170]. Remangilones A and C, which are oleanane triterpenoids isolated from *Physena madagascariensis*, exhibit cytotoxicity against two breast cancer cell lines, MDA-MB-231 and MDA-MB-435, and induce apoptosis [171]. Amooranin (AMR), a triterpene acid isolated from the tropical tree *Amoora rohituca*, was shown to have cytotoxicity against MCF-7 cells [172]. Also, in studies of the mechanism of AMR-related cell death, AMR was reported to induce apoptosis through elevating caspase activity in MCF-7 and multidrug resistant MCF-7/TH cells, to suppress cell growth by arresting the cell cycle, and to induce apoptosis by regulating Bcl-2 family proteins and caspases in MDA-468 and MCF-7 cells [173,174]. Tirucallane-type triterpenoids extracted from *Amphipterygium adstringens* had cytotoxic effects on MCF-7 cells [175]. A newly discovered triterpenoid, Ailanthus excelsa chloroform extract-1 (AECHL-1) from *Ailanthus excelsa* Roxb, was shown to regress tumor volume in nude mice injected with MCF-7 cells [176].

Recently, curcumin has been also studied as an inhibitor of breast cancer cell proliferation. It was found to prevent the proliferation of Bisphenol A (BPA) induced MCF-7 cells by suppressing BPA-upregulated expression of miRNA-19, a key oncogenic miRNA [177]. Resveratrol and resveratrol sulfates reduced the cell viability of breast cancer cells (MCF-7, ZR-75-1, and MDA-MB-231) [178]. Avicennia marina extracts, used in traditional medicine, were shown to induce apoptosis in breast cancer cells (AU565, MDA-MB-231, and BT483) and inhibit tumor growth in MDA-MB-231 transplanted nude mice [179]. Additionally, these extracts were found to be rich in polyphenols [179]. In a recent study, hydroxycinnamic acid and flavonol derivatives, present in Bursera copallifera, were shown to be involved in inhibiting the migration of MCF-7 and MDA-MB-231 cells [180]. As in the case of colorectal cancer, dichamanetin also reduced the cell viability of MDA-MB-231 cells [80].

### 3.5. Prostate Cancer

Prostate cancer is one of the most commonly diagnosed cancers in men worldwide. Diet and lifestyle are thought to be major contributors to prostate cancer development, and therefore, the ability of bioactive natural plant chemicals to inhibit prostate cancer has been widely studied [181]. Recently, decursinol, a metabolite of *Angelica gigas*, has been shown to decrease tumor growth in mice with xenografts of human DU145 and PC3 prostate cancer cells [182], and another group has reported that decursin and decursinol angelate (DA) from *Angelica gigas* Nakai (AGN) have inhibitory effects on the growth of prostate epithelium in the transgenic adenocarcinoma of mouse prostate (TRAMP) model [183]. Both resveratrol and γ-viniferin, a tetramer of resveratrol, inhibit the growth of LNCaP prostate cancer cells by arresting the cell cycle at the G1 phase; γ-viniferin has more potent growth-inhibiting activity than resveratrol [184]. Another plant polyphenol, fisetin, has been found to be involved in regulating microtubule stability through increasing the amount of acetylated α-tubulin and microtubule associated proteins (MAP)-2 and 4 in PU3 and DU145 cells and downregulating nuclear migration protein (NudC), which plays an essential role in mitosis and cytokinesis [185]. Prostate-cancer-associated mortality is mainly caused by metastasis. Therefore, it is important to develop anti-cancer compounds to inhibit its metastasis. Genistein was found to act as an anti-metastatic agent to inhibit cellular invasion in prostate cancer cells through decreasing MMP expression and decreasing the formation of metastases in mice implanted with the PC3-M human prostate cancer cell line [186]. Curcumin was discovered to inhibit cancer-associated fibroblast (CAF)-induced EMT and invasion in PC3 cells by suppressing the monoamine oxidase A (MAOA)/mTOR/HIF-1α signaling pathway [187]. Additionally, it has anti-cancer effects through the inhibition of prostate cancer cell growth and metastasis [188,189]. Both SFN and I3C attenuate Akt/NKκB signaling and induce growth arrest and apoptosis in prostate cancer [181].

### 3.6. Hematologic Cancer

Hematologic cancer, also called blood cancer, develops in blood-forming tissue or in immune-system-related cells and includes leukemia, myeloma, and lymphoma [190]. Its overall prognosis is poor despite extensive research into cytotoxic agents to combat it. Recently, hypericin, a secondary metabolite from *Hypericum* (Saint John’s wort), was discovered to potentiate the mitoxantrone (MTX)-induced death of the HL-60 subclone human leukemia cells, in which the ABC transporter is overexpressed [191]. The anti-cancer mechanisms of the natural polyphenol resveratrol have been widely studied. Azmi et al. showed that resveratrol induces DNA breakage in the presence of copper in human peripheral lymphocytes, suggesting a novel anti-cancer mechanism involving the mobilization of endogenous copper, which is known to be increased in various malignancies [192]. Another group discovered that resveratrol inhibited cell proliferation, arrested the cell cycle in the S-phase, and induced apoptosis in the acute myeloid leukemia cells OCI-ANK3 and OCIM2 [193]. Similarly to in lung cancer, EGCG also induced the apoptotic death of the human B lymphoblastoid cell line (Ramos cells) in a dose- and time-dependent manner [194]. In addition, I3C was found to have anticancer properties in B cell precursor acute lymphoblastic leukemia in NALM-6 cells. It caused the arrest of the G1 phase in cell cycle and triggered apoptosis [195].

### 3.7. Skin Cancer

Skin cancer is one of the tumors causing malignancies around the world, and its incidence is increasing alarmingly [196]. Skin cancer is believed to develop through co-carcinogenic effects, and many natural metabolites have been widely studied as anti-carcinogens. In particular, allyl sulfides including diallyl sulfide (DAS), diallyl disulfide (DADS), and diallyl trisulfide (DATS) have been reported to prevent the progression of skin cancer [197]. DAS was found to have anti-mutagenic properties against 7,12-dimethylbenz[a]anthracene (DMBA), a carcinogenic polycyclic aromatic hydrocarbon that induces DNA strand breaks in mouse skin [198]. Additionally, DAS induced apoptosis in DMBA-mediated mouse skin tumors through multiple mechanisms, including the up-regulation of tumor suppressor protein p53, its downstream proteins, and proapoptotic proteins such as Bax, and the reduction of Ras onco-protein expression [199,200]. Pomolic acid, a triterpenoid found in *Polylepis* *racemosa,* was reported to have cytotoxic effects on M-14 melanoma cells [167].

### 3.8. Head and Neck Cancer

Head and neck cancer is one of the leading causes of death worldwide [201]. Current medical and surgical treatments for these malignancies result in functional morbidity and side effects; thus, chemopreventive phytochemicals have been widely studied [201]. β-Carotene is one of the most abundant carotenoids, which are natural pigments found in plants and that are well known to be effective antioxidants [202]. Recently, β-carotene has been reported to enhance the inhibitory effect of 5-FU, a medication used against cancer, on tumor growth of xenografts of Eca109 esophageal squamous cell carcinoma (ESCC) cells in nude mice and to inhibit cell proliferation in the ESCC cells EC1 and Eca109 [203]. EGCG has been found to have cytotoxic effects via arrest of the cell cycle at G1 and the induction of apoptosis in the human head and neck squamous cell carcinoma (HNSCC) cell lines YCU-N861 and YCU-H891 [204]. It has also been reported to synergistically inhibit the growth of HNSCC cells via inhibition of the NF-κB signaling pathway when used in conjunction with erlotinib, a tyrosine kinase inhibitor of EGFR, which is frequently overexpressed in HNSCC cells [205]. In addition, EGCG was shown to inhibit the invasion and migration of the human oral cancer cell line OC2 through decreasing MMP-2, -9, and uPA in a dose dependent manner without cytotoxicity [206].

## 4. Conclusions and Perspectives

Up to the present date, several thousands of different metabolites have been identified in plants and studied for their effectiveness in a wide variety of applications [6]. We have categorized plant-derived metabolites into several major classes based on their structure, and the structural characteristics of each class were discussed. Also, natural compounds with anti-cancer activity were summarized according to type of cancer (Table 1 and Table 2). Medicinal plants have been used since ancient times, and are still used as a primary source of medical treatment in developing countries [3]. Plant-derived substances have advantages including their low cost and the rapid speed of discovery of new drugs; their main disadvantage is the absence of common international standards for evaluating their quality, efficacy, and safety [3]. Additionally, the incidence of various malignant cancers has been growing, and conventional cancer therapies have limitations, including the high toxicity and side effects of anti-cancer drugs [3]. For this reason, a broad multidisciplinary research approach involving ethnopharmacology, botany, pharmacognosy, and phytochemistry is required for the successful application of phytochemicals in the treatment or prevention of cancer [207]. Also, the biotechnological production of secondary metabolites of naturally occurring plant substances and the combination of phytochemicals with existing anti-cancer drugs or other chemical compounds represent alternative approaches to natural-product-based drug development. Furthermore, besides the cytotoxic effects of plant metabolites, additional therapies that treat cancers by different mechanisms are required for the development of new drugs from plant metabolites. One of the new cancer treatment method focuses on the immunomodulation of the tumor microenvironment. Therefore, the development of natural-product-based drugs that can regulate the functioning of the immune system in the tumor microenvironment will be a novel cancer treatment option in the future. This review provides comprehensive information on the various classes of plant-derived metabolites and bioactive plant compounds that have shown anti-cancer activity in vitro or in vivo models of different types of cancer. The data we have summarized clearly suggests that natural metabolites from plants play a major role as the most prominent source of anti-cancer treatments.

## Figures and Tables

**Table 1:** Structural classification of active metabolites with anticancer activity.

Class	Active Metabolite	Structure
**Phenolic compounds**	Curcumin	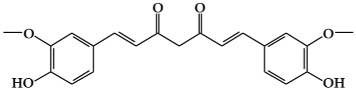
	Decursin	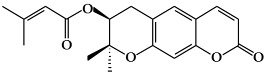
	Decursinol	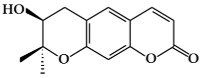
	Decursinol angelate	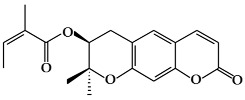
	Dichamanetin	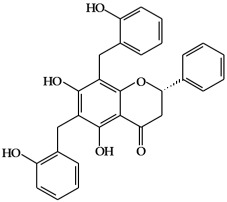
	Epigallocatechin gallate (EGCG)	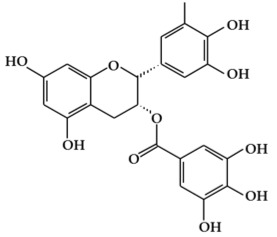
	Fisetin	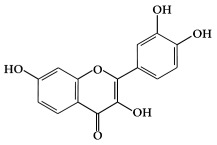
	Genistein	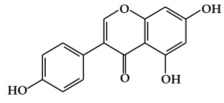
	Hydroxycinnamic acid	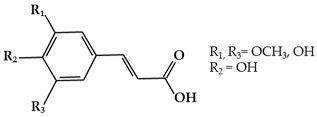
	Hypericin	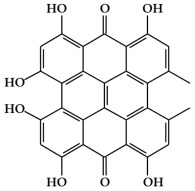
	Quercetin	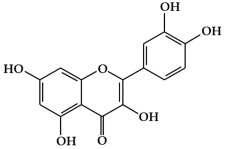
**Phenolic compounds**	Resveratrol	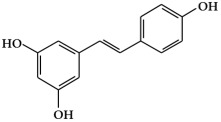
	Punicalagin (PC)	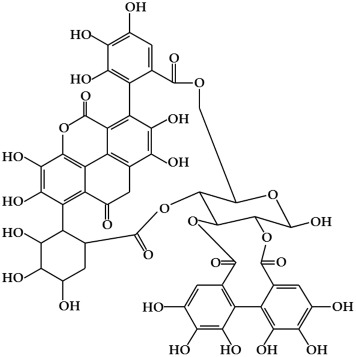
	γ-viniferin	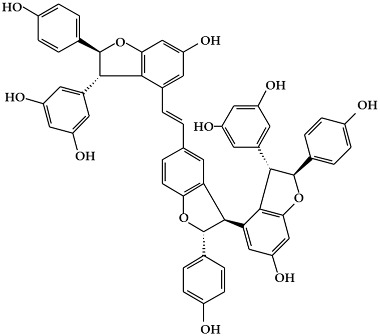
**Terpenoids**	Asiatic acid	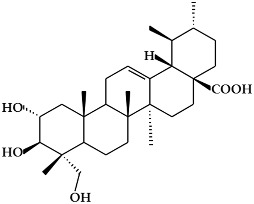
	Ailanthus excelsa chloroform extract-1 (AECHL-1)	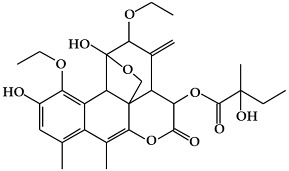
	Amooranin (AMR)	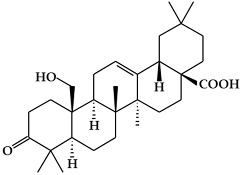
	Auraptene (AUR)	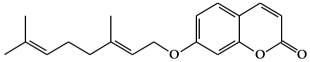
	Betulinic acid (BA)	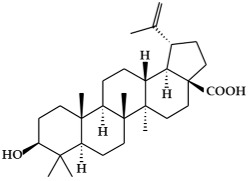
	Celastrol	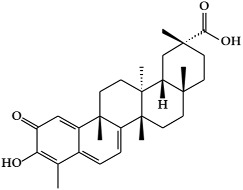
	Curcubitane-type triterpenoids (Balsaminapentaol)	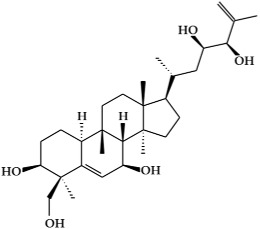
	Dammarane triterpenoid (Cabraleadiol)	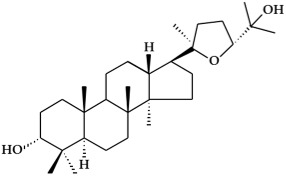
	Lupeol	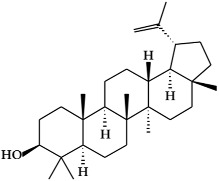
	Meliavolkenin	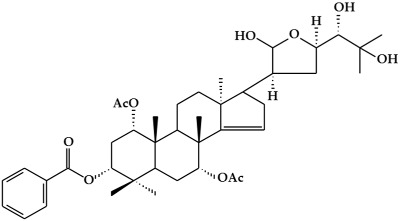
	Pomolic acid	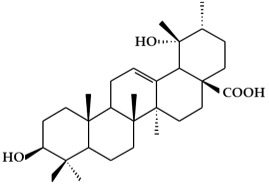
	Pristimerin	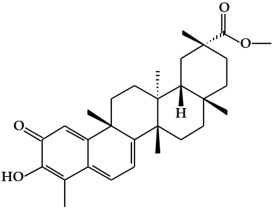
	Remangilones A	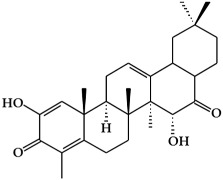
	Remangilones C	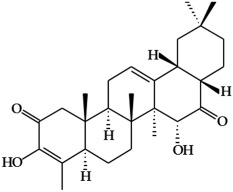
	Tirucallane-type triterpenoids	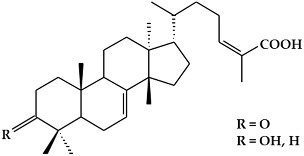
	Ursolic acid	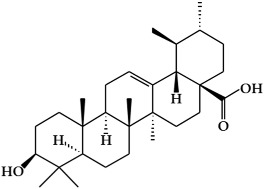
	β-carotene	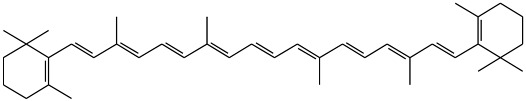
**Nitrogen-containing alkaloids & sulfur-containing compounds**	Allicin	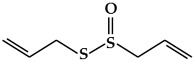
	Benzyl isothiocyanate (BITC)	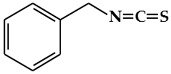
	Diallyl sulfide (DAS)	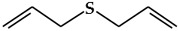
	Indole-3-carbinol (I3C)	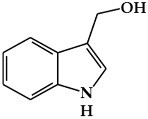
	Phenethyl isothiocyanate (PEITC)	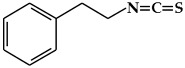
	Sulforaphane (SFN)	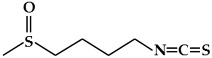
	Tomatine	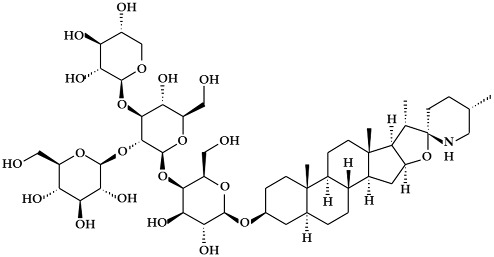
	6-MSITC	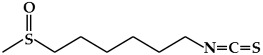

**Table 2:** Anti-cancer effects of active metabolites from plants in different types of cancer.

Type of Cancer	Active Metabolites	In Vitro or In Vivo Effects	IC_50_ & Effective Concentration (EC) (μM).	Ref.
**Colorectal cancer**	Dichamanetin	Induction of ROS and cell cycle arrest in HT-29 colon cancer cells	IC_50_: 13.8	[80]
	GAP	Suppression of colon carcinogenesis in DSS mice	EC: 0.01 % or 0.05 % in diet	[83]
	Auraptene	Inhibition of the growth of colon cancer cells and suppression of colonosphere formation	EC: 10	[85]
	Curcumin	Suppression of the oncogenicity of human colon cancer cells and the growth of HCT-116 tumor xenografts	EC: 10	[87]
	Genistein	Inhibition of cell proliferation and induction of apoptosis in HCT 116 and LoVo cells	EC: 135	[89,90,91]
		Inhibition of metastasis in colorectal cancer cell implanted nude mice		
	I3C+Genistein	Induction of apoptosis in HT 29 colon cancer cells	I3C EC: 300 Genistein EC: 40	[92]
	BITC	Suppression of viability in HCT 116 colon cancer cells	EC: 5–20	[93]
	SFN	Induction of apoptosis and inhibition of proliferation in HT 29 colon cancer cells	EC: 5–20	[94]
	PEITC	Reduction of colon carcinogenesis in AOM/DSS induced mice	EC: 0.12 % in diet	[95]
	6-MSITC	Induction of apoptosis in HCT 116 colon cancer cells	IC_50_: 0.92–10.01	[96]
**Gastric cancer**	Resveratrol	Inhibition of proliferation in gastric cancer cells	EC: 50–200	[99]
	SFN	Prevention of benzo[a]pyrene-induced stomach tumors in mice	EC: 1.33 mg per mouse	[101]
	PEITC	Induction of cell cycle arrest and apoptosis in gastric cancer cells MKN74 and Kato-III	EC: 17.8	[102,103]
		Inhibition of migration and invasion in AGS gastric cancer cells		
	BITC	Inhibition of migration and invasion in AGS gastric cancer cells	EC: 0.25–0.5	[104]
	Curcumin	Inhibition of proliferation in SGC-7901 gastric cancer cells	EC: 15–60	[105,106]
		Reduction of xenograft tumor growth in mice		
		Reduction of LVD in gastric cancer bearing nude mice		
	Quercetin	Induction of apoptosis in BGC-823 gastric cancer cells	EC: 30–120	[107]
	Allicin	Inhibition of gastric cancer cell growth	EC: 184.88	[109]
**Lung cancer**	EGCG	Induction of cell cycle arrest and apoptosis in lung cancer cells Reduction of proliferation and growth in lung cancer cells Inhibition of TGF-β-induced cell migration, invasion, and EMT in NSCLC cells	IC_50_: 70	[112,113,114]
	BITC	Inhibition of growth in A549 lung cancer cells	EC: 10	[115,116,117]
		Inhibition of tumorigenesis in PAH-induced A/J mice		
	PEITC	Induction of apoptosis in NSCLC cells	EC: 12.5–20	[113,118]
	SFN	Induction of apoptosis in NSCLC cells	EC: 10	[119,120]
		Alleviation of carcinogenic lung in B(a)P induced lung cancer bearing mice		
	I3C	Induction of apoptosis in A549 lung adenocarcinoma cells in combination with TRAIL	EC: 100–500	[121,122,123,124]
		Inhibition of progression of tobacco carcinogen induced lung adenocarcinoma progression		
		Induction of cell cycle arrest and apoptosis in A549 lung cancer cells		
		Inhibition of NNK-induced lung tumors in combination with silibinin in mice		
	Genistein	Inhibition of carcinogenesis in mice with VC-induced lung cancer Inhibition of cell proliferation and induction of apoptosis in H446 SCLC cells	IC_50_: 81	[125,126,127]
		Inhibition of cell proliferation and induction of apoptosis in combination with gefitinib in H1975 NSCLC cells		
	Fisetin	Inhibition of cell growth and induction of apoptosis in combination with chemotherapeutic agents in H460 NSCLC cells	IC_50_: 59	[129,130,131,132]
		Inhibition of cell viability and colony-forming activity in A549 NSCLC cells		
		Inhibition of the invasion and migration of A549 NSCLC cells		
		Inhibition of lung carcinogenesis in B(a)P-induced mice		
		Inhibition of angiogenesis and tumor growth in Lewis lung carcinoma bearing mice		
	Punicalagin	Anti-proliferative effects on A549 and H1299 NSCLC cells Inhibition of tumor growth in mice with xenografts of A549 NSCLC cells Inhibition of B(a)P-induced tumorigenesis in A/J mice	EC: 11.52–184.3	[134,135,136]
	Curcumin	Inhibition of cell growth and invasion in NSCLC cells Lowering the resistance of NSCLC cells against erlotinib Suppression of cell proliferation, the cell cycle, migration, invasion, and angiogenesis in SCLC cells Induction of apoptosis in SCLC cells	EC: 30	[137,138,139,140,141]
**Breast cancer**	Tomatine	Induction of cell cytotoxicity and apoptosis in MCF-7 breast cancer cells	IC_50_: 7.07	[144]
	I3C	Increasing apoptotic cell death and decreasing the proliferation of the ERα-positive breast cancer cells Disruption of in vitro 10AT-Her2 cell tumorsphere formation and in vivo tumor xenograft growth	IC_50_: 204	[145,146]
	Curcubitane-type triterpenoids	Inhibition of cell growth and induction of apoptosis in human breast cancer cells	EC: 0.5–35.7	[148,149,150,151,152,153,154,155]
	Dammarane triterpenoids	Cytotoxicity against breast cancer cells	EC: 20.97	[156]
	Pristimerin	Induction of apoptosis in MDA-MB-231 breast cancer cells	EC: 1–3	[157]
	Celastrol	Inhibition of cell growth and invasion and induction of apoptosis in W256 breast cancer cells	EC: 1	[158]
	Meliavolkenin	Cytotoxicity against MCF7 breast cancer cells	EC: 6.05	[159]
	Betulinic acid	Induction of anti-proliferation in MCF7 and T47D breast cancer cells	IC_50_: 2.4	[160,161,162]
	Lupeol	Inhibition of MDA-MB-231 ERα-negative cell proliferation	EC: 1–30	[163]
	Ursolic acid	Inhibition of proliferation and induction of apoptosis in MCF7 cells Suppression of migration and invasion in MDA-MB-231 cells	IC_50_: 3.26	[164,165,166,167,168,169]
	Asiatic acid	Inhibition of cell growth and induction of apoptosis in MCF7 and MDA-MB 231 cells	IC_50_: 5.95–8.12	[170]
	Remangilones A and C	Cytotoxicity against MDA-MB-231 and MDA-MB-435 cells	RemangilonesA IC_50_: 6.6–8.5 RemangilonesC IC_50_: 1.6–2.0	[171]
	Amooranin	Induction of apoptosis and suppression of cell growth in MDA-468 and MCF7 cells	IC_50_: 3.82-7.22	[172,173,174]
	Tirucallane-type triterpenoids	Cytotoxicity against MCF7 cells	IC_50_: 41.33–86.14	[175]
	AECHL-1	Regression of MCF7 xenograft tumors in nude mice	EC: 5–100	[176]
	Curcumin	Anti-proliferation of BPA-induced MCF7 cells	EC: 1	[177]
	Resveratrol	Reduction of cell viability in breast cancer cells (MCF-7, ZR-75-1, and MDA-MB-231)	IC_50_: 67.6–82.2	[178]
	Hydroxycinnamic acid	Inhibition of migration in MCF-7 and MDA-MB-231 cells	IC_50_: 75.71	[180]
	Dichamanetin	Induction of ROS and cell cycle arrest in MDA-MB-231 cells	EC: 8.7	[80]
**Prostate cancer**	Decursinol	Suppression of tumor growth in mice with xenografted DU145 and PC3 prostate cancers	EC: 4.5 mg per mouse	[182]
	Decursin & Decursinol angelate	Inhibition of prostate epithelium growth in the TRAMP model	EC: 3 mg per mouse	[183]
	Resveratrol & γ-viniferin	Inhibition of the growth of LNCaP prostate cancer cell	Resveratrol IC_50_: 10.23-228.3 γ-viniferin IC_50_: 8.93–90.1	[184]
	Fisetin	Inhibition of cell growth and proliferation in PU3 and DU145 cells	EC: 20–80	[185]
	Genistein	Inhibition of cellular invasion in in vitro prostate cancer and in vivo metastasis formation in mice with xenografts of PC3-M prostate cancer	EC: 10	[186]
	Curcumin	Inhibition of CAF-induced EMT and invasion in PC3 cells Induction of cell cycle arrest and apoptosis in in vitro prostate cancer cells and the in vivo TRAMP model	EC: 25	[187,188,189]
	SFN and I3C	Induction of cell cycle arrest and apoptosis of PC3, LNCaP, and DU145 cells in vitro	SFN EC: 40 I3C EC: 30–100	[181]
**Hematologic cancer**	Hypericin	Attenuation of MTX cytotoxicity in HL-60 promyelocytic leukemia cells	EC: 0.1–0.5	[191]
	Resveratrol	Induction of DNA breakage in human peripheral lymphocytes Induction of apoptosis in OCI-ANK3 and OCIM2 acute myeloid leukemia cells	EC: 10–75	[192,193]
	EGCG	Induction of apoptotic death in Ramos B lymphoblastoid cells	EC: 60–100	[194]
	I3C	Inhibition of cell growth and induction of apoptosis in pre-B acute lymphoblastic leukemia cells	EC: 60	[195]
**Skin cancer**	Diallyl sulfide	Reduction of DNA strand breaks in DMBA induced mouse skin Induction of apoptosis in DMBA-induced mouse skin tumors	EC: 25	[197,198,199,200]
	Pomolic acid	Cytotoxic effects against M-14 melanoma cells	EC: 14.6	[167]
**Head and neck cancer**	β-carotene	Inhibition of tumor growth in nude mice with xenografts of Eca109 ESCC cell xenografts	EC: 30	[203]
	EGCG	Induction of cell cycle arrest and apoptosis in YCU-N861 and YCU-H891 HNSCC cells Inhibition of cell growth in combination with erlotinib in HNSCC cells Inhibition of the invasion and migration in oral cancer cell OC2	EC: 30–60	[204,205,206]

## Abbreviations

OSC	Organosulfur compounds
GOFA	4′-geranyloxyferulic acid
GAP	3-(4′-geranyloxy-3′;-methoxyphenyl)-l-alanyl-l-proline
AOM	Azoxymethane
DSS	Dextran sodium sulfate
β-CD	β-cyclodextrin
AUR	Auraptene
SIRT1	Sirtuin 1
ITCs	Isothiocyanates
SFN	Sulforaphane
BITC	Benzyl isothiocyanate
PEITC	Phenethyl isothiocyanate
MMPs	Matrix metalloproteinases
LVD	Lymphatic vessel density
EGCG	Epigallocatechin gallate
miRNA	micro RNA
HIF-1α	Hypoxia-inducible factor 1alpha
TGF-β	Transforming growth factor β
EMT	Epithelial-to-mesenchymal transition
ERK1/2	Extracellular signal–regulated kinases 1/2
NSCLC	Nonsmall cell lung cancer
SCLC	Small cell lung cancer
MAPK	Mitogen-activated protein kinase
B(a)P	Benzo(a)pyrene
TRAIL	Tumor necrosis factor-related apoptosis-inducing ligand
I3C	Indole-3-carbinol
VC	Vinyl carbamate
EGFR	Epidermal growth factor receptor
NF-κB	Nuclear factor kappaB
mTOR	mammalian target of rapamycin
u-PA	urokinase-type plasminogen activator
CPA	Cyclophosphamide
PC	Punicalagin
PFE	Pomegranate fruit extracts
MTA1	Metastasis-associated protein1
AMPK	AMP-activated protein kinase
STAT3	Signal transducer and activator of transcription 3
ROS	Reactive oxygen species
ERα	Estrogen receptor α
AhR	Aryl hydrocarbon receptor
ATF-3	cyclic AMP dependent transcription factor
IKK	Inhibitor against IκB kinase
BA	Betulinic acid
GR	Glucocorticoid receptor
AP1	Activator protein 1
JNK	c-Jun N-terminal Kinase
AMR	Amooranin
AECHL-1	Ailanthus excelsa chloroform extract-1
BPA	Bisphenol A
miRNA-19	microRNA-19
AGN	Angelica gigas Nakai
TRAMP	Transgenic adenocarcinoma of mouse prostate
MAP	Microtubule associated proteins
NudC	Nuclear migration protein
CAF	Cancer associated fibroblast
MAOA	Monoamine oxidase A
MTX	Mitoxantrone
DAS	Diallyl sulfide
DADS	Diallyl disulfide
DATS	Diallyl trisulfide
DMBA	7,12-dimethylbenz[a]anthracene
ESCC	Esophageal squamous cell carcinoma
HNSCC	Head and neck squamous cell carcinoma
